# Induction therapy with cetuximab plus docetaxel, cisplatin, and 5-fluorouracil (ETPF) in patients with resectable nonmetastatic stage III or IV squamous cell carcinoma of the oropharynx. A GERCOR phase II ECHO-07 study

**DOI:** 10.1002/cam4.408

**Published:** 2015-02-14

**Authors:** Benoist Chibaudel, Roger Lacave, Marine Lefevre, Patrick Soussan, Martine Antoine, Sophie Périé, Jean-Baptiste Belloc, Alain Banal, Sébastien Albert, Frédéric Chabolle, Philippe Céruse, Philippe Baril, Michel Gatineau, Martin Housset, Rachel Moukoko, Magdalena Benetkiewicz, Aimery de Gramont, Franck Bonnetain, Jean Lacau St Guily

**Affiliations:** 1 Division of Medical Oncology, Franco-British Hospital InstituteLevallois-Perret, France; 2 GERCOR (Cooperator Multidisciplinary Oncology Group)Paris, France; 3 GERCOR-IRC (GERCOR-Innovative Research Consortium)Paris, France; 4 Department of Histology and Tumor Biology, Hospital TenonParis, France; 5 ER2 Division, University Pierre et Marie CurieParis, France; 6 Clinical Research Group (GRC), Hospital TenonParis, France; 7 Division of Anatomic Pathology, Hospital TenonParis, France; 8 Unit of Virology, Hospital TenonParis, France; 9 Department of Otorhinolaryngology-Cervicofacial Surgery, Hospital TenonParis, France; 10 Department of Otorhinolaryngology-Maxillofacial Surgery, Hospital Simone VeilMontmorency, France; 11 Department of Otorhinolaryngology-Head and Neck Surgery, Centre René HugueninSaint-Cloud, France; 12 Department of Otorhinolaryngology-Head and Neck Surgery, Hospital Bichat-Claude BernardParis, France; 13 Department of Otolaryngology and Cervicofacial Surgery, Hospital FochSuresnes, France; 14 Department of Otorhinolaryngology-Cervicofacial Surgery, Hospital center Lyon-SudLyon, France; 15 Department of Otorhinolaryngology-Head and Neck Surgery, Hospital DelafontaineSaint-Denis, France; 16 Medical Oncology Service, Groupe Hospitalier Saint JosephParis, France; 17 Department of Radiation Oncology, Georges Pompidou European HospitalParis, France; 18 Methodological and Quality of Life in Oncology Unit (EA3181) & Quality of Life and Cancer Clinical Research Platform, Besançon University HospitalBesançon, France

**Keywords:** Cetuximab, chemotherapy, induction therapy, oropharyngeal cancer, papillomavirus

## Abstract

Induction TPF regimen is a standard treatment option for squamous cell carcinoma (SCC) of the oropharynx. The efficacy and safety of adding cetuximab to induction TPF (ETPF) therapy was evaluated. Patients with nonmetastatic resectable stage III/IV SCC of the oropharynx were treated with weekly cetuximab followed the same day by docetaxel and cisplatin and by a continuous infusion of 5-fluorouracil on days 1-5 (every 3 weeks, 3 cycles). The primary endpoint was clinical and radiological complete response (crCR) of primary tumor at 3 months. Secondary endpoints were crCR rates, overall response, pathological CR, progression-free survival, overall survival, and safety. Forty-two patients were enrolled, and 41 received ETPF. The all nine planned cetuximab doses and the full three doses of planned chemotherapy were completed in 31 (76%) and 36 (88%) patients, respectively. Twelve (29%) patients required dose reduction. The crCR of primary tumor at the completion of therapy was observed in nine (22%) patients. ETPF was associated with a tumor objective response rate (ORR) of 58%. The most frequent grade 3–4 toxicities were as follows: nonfebrile neutropenia (39%), febrile neutropenia (19%), diarrhea (10%), and stomatitis (12%). Eighteen (44%) patients experienced acne-like skin reactions of any grade. One toxic death occurred secondary to chemotherapy-induced colitis with colonic perforation. This phase II study reports an interesting response rate for ETPF in patients with moderately advanced SCC of the oropharynx. The schedule of ETPF evaluated in this study cannot be recommended at this dosage.

## Introduction

Cancer of the upper aerodigestive tract, predominantly squamous cell carcinoma (SCC) of the oropharynx, is the fifth most common malignancy and the seventh leading cause of cancer death in France 1. The majority of diagnosed patients present locally advanced disease invading underlying structures and/or spreading to regional lymph nodes (stages III and IV). The survival rates are relatively poor ranging from 30% to 60% at 5 years 2.

The standard treatment in moderately advanced disease (i.e., resectable) includes surgery with appropriate adjuvant therapy, and chemoradiotherapy in patients with advanced disease (i.e., unresectable) 3. Induction therapy with cisplatin prior to definitive chemoradiotherapy is still controversial 4,5.

The most active induction chemotherapy regimen in patients with unresectable SCC of the oropharynx is the combination of 5-fluorouracil (5-FU), docetaxel, and cisplatin (TPF) 6,7. However, an improvement in survival comparing induction therapy followed by chemoradiotherapy to direct chemoradiotherapy has not yet been established.

An overexpression of the epidermal growth factor receptor (EGFR) or any of its linked pathways occurs in more than 90% of head and neck SCC 8. Increased EGFR protein expression or *EGFR* gene copy number amplification are associated with poor prognosis 8–10, radiation-resistance 11, locoregional treatment failure 10, and increased rates of distant metastases 10,12. Monoclonal antibody cetuximab blocks ligand-induced EGFR activation 13 and improves survival when used concurrently in combination with radiotherapy in locoregionally advanced disease 14 and cisplatin/5-FU-based chemoradiotherapy in recurrent/metastatic setting 15.

Human papillomavirus (HPV) type-16 (HPV16) infection has been associated with an increased risk of developing oropharyngeal cancer 16. In contrary to the HPV-negative tumors (primarily related to tobacco use and alcohol consumption) 17,18, an increasing incidence and greater responsiveness to radiotherapy of HPV-positive tumors have been reported 19–21. The potential role of anti-EGFR treatment in HPV16-positive locally advanced oropharyngeal SCC cancer remains questionable 22.

The aim of this study was to evaluate the cetuximab-TPF combination (ETPF) as induction therapy in treatment of patients with locally advanced resectable SCC of the oropharynx.

## Materials and Methods

### Study design

ECHO-07 (ClinicalTrials.gov #NCT00665392) was a prospective multicenter single-arm open-label phase II study. The protocol was approved by the National Security Agency for Medicines and Health Products (ANSM, France) and Ethics Committee of Groupe Hospitalier Pitié-Salpétrière (Paris VI, France). All patients provided written informed consent.

### Patient eligibility criteria

Eligible patients were 18–75 years with previously untreated, resectable AJCC/UICC TNM (American Joint Committee on Cancer/Union Internationale Contre le Cancer) stage III (T3/T1-2N1-2M0) to IVB (T4/T1-3N3M0) SCC of the oropharynx 23. Other eligibility criteria included measurable or evaluable disease (Response Evaluation Criteria in Solid Tumors [RECIST] 1.0), an Eastern Cooperative Oncology Group (ECOG) performance status (PS) of 0–1, adequate laboratory parameters (absolute neutrophil count (ANC) ≥1500/mm^3^, platelet count ≥100,000/mm^3^, hemoglobin ≥9 g/dL, creatinine <1.5-fold the upper limit of the normal (ULN) value) and no uncontrolled cardiac or other disease.

### Induction chemotherapy

Treatment consisted of cetuximab by intravenous (IV) infusion over 1–2 h on days 1, 8, and 15 (loading dose of 400 mg/m^2^ on day 1, then 250 mg/m^2^ weekly) followed the same day by docetaxel and cisplatin both given as a 1h IV infusion (at a 75 mg/m^2^ dose) and by 5-FU IV infusion on days 1–5 (at a 750 mg/m^2^ dose per day). Treatment was given every 3 weeks for a maximum of three cycles. Pre- and concomitant medication consisted of IV hydration and infusion of diphenhydramine hydrochloride and dexamethasone. A primary prophylaxis with granulocyte colony stimulating factors (G-CSF) was required.

### Response assessments

Baseline assessment including medical history, physical examination, otolaryngology evaluation with nasofibroscopy, laboratory evaluation, histological diagnosis, and computed tomography (CT) scan of the neck and chest was performed within 3 weeks prior to induction therapy initiation. During ETPF treatment, patients were assessed for toxicity before each cycle of chemotherapy. The evaluation of tumor response was assessed at 3 months from inclusion and before local treatment using clinical examination and RECIST 1.0 criteria. After local treatment, patients were evaluated regularly for 3 years.

The primary endpoint was clinical and radiological complete response (crCR) rate of primary tumor. Secondary endpoints were clinical complete response (cCR) rate, radiological complete response (rCR) rate, overall survival (OS), progression-free survival (PFS), pathological complete response (pCR), safety, and biomarkers analysis. OS was defined as the time interval between patient inclusion and death (all causes). Patients for whom death was not recorded were censored at the date of last news. PFS was defined as the time interval from inclusion to the first local, regional and/or distant progressive disease (PD), or death (all causes). Alive patients without PD were censored at the date of last news.

Adverse events (AE) were collected during induction treatment and follow-up visits. Toxicity evaluation was carried out according to the National Cancer Institute Common Terminology Criteria for Adverse Events (NCI-CTCAE, v3.0) scale.

### Postinduction therapy

Local treatment with surgery or chemoradiotherapy after induction therapy was not part of the study protocol and was performed at investigator's discretion.

### Biomarkers analysis

For each patient, pretreatment formalin-fixed, paraffin-embedded (FFPE) tumor tissue blocks or FFPE unstained slides of primary tumor and cryopreserved tumor blocks for molecular analysis of the EGFR pathway components and HPV genotyping were required. The potential predictive value of EGFR-related biomarkers for response to ETPF induction therapy was evaluated by (1) EGFR gene and EGFR ligands encoding genes expression analyses (epidermal growth factor [EGF], transforming growth factor *α* [TGF*α*], amphiregulin [AREG], epiregulin [EREG], heparin-binding EGF-like growth factor [HB-EGF], and betacellulin [BTC]) performed by semiquantitative real-time-PCR 24, (2) EGFRvIII gene expression analysis performed according to Sok et al. 25, (3) EGFR-intron 1 polymorphism analysis according to Etienne-Grimaldi et al. 26, and (4) EGFR gene copy-number assessed by fluorescent in situ hybridization (FISH) using an EGFR/CEN-7 FISH DNA/PNA probe (Dako, France). The high-risk HPV16 genotype screen (by PCR) and quantification of HPV16 viral DNA (by RT-PCR) were assessed.

### Statistics

Given that a complete response (CR) rate ≤10% was unsatisfactory, a CR rate ≥30% was expected. To test the efficacy and safety of the treatment, 40 evaluable patients were required to reach a power of 90% and at a significance level 5% (a one-sided type I error). Assuming 5% nonevaluable patients, a total of 42 patients had to be enrolled. Analyses were performed on a modified intent-to-treat (mITT) population (patients were considered evaluable for tumor response if they had received at least one dose of ETPF combination). Means (min-max) and standard deviations (SDs) were used to describe continuous variables; categorical variables were expressed in terms of frequencies and percentages together with 95% confidence intervals (CIs). Response rates and corresponding 95% CIs were calculated using a binomial distribution. Survivals and median follow-up were estimated using the Kaplan–Meier and reverse Kaplan–Meier methods, respectively. Stratified hazards ratios (HRs) were calculated using the univariate Cox proportional hazard model. Correlational research of EGFR-related biomarkers and HPV status in tumors and blood samples obtained prior and after induction therapy were done for exploratory purpose as planned in the study protocol. Statistical analyses were performed using SAS 9.1 software (SAS Institute Inc, Cary, NC).

## Results

### Patient characteristics

Between July 2008 and November 2011, 42 patients 42 patients were enrolled from nine centers (Table1). Median age of patients was 56 years, with 81% males. The majority of patients (79%) were ECOG PS 0, had a primary tumor located in the tonsil area (88%) and a stage III disease (76%).

**Table 1:** Patient characteristics at baseline of the total study population (*n* = 42)

Characteristics	*N*	%
Sex		
Male	34	81.0
Female	8	19.0
Age in years, mean ± SD	56.1 ± 6.8	
ECOG performance status		
0	33	78.6
1	8	19.0
Missing	1	2.4
Grade of differentiation		
Well	17	40.5
Moderate	18	42.9
Poor or undifferentiated	4	9.5
Missing	3	7.1
Primary tumor localization		
Anterior	3	7.1
Lateral (tonsil area)	37	88.1
Posterior	1	2.4
Superior	1	2.4
Node involvement		
Group I	1	2.4
Group IIa	31	73.8
Group IIb	9	21.4
Group III	10	23.8
Group IV	0	–
Group V	4	9.5
Group VI	0	–
T-stage		
T2	13	31.0
T3	24	57.1
T4	5	11.9
N-stage		
N0	5	11.9
N1	9	21.4
N2	27	64.3
N3	1	2.4
Staging		
III	32	76.2
IV	10	23.8
Lip mobility		
Normal	40	95.2
Decreased	2	4.8
Trismus		
Yes	5	11.9
No	37	88.1
Creatinine clearance (mL/min)		
<60	1	2.4
60–120	31	73.8
>120	8	19.1
Albuminemia (g/L)		
<40	8	19.1
≥60	14	33.3
Missing	20	47.6
Life style risk factors		
Alcohol	3	7.1
Tobacco	8	19.0
Alcohol + tobacco	25	59.5
None	6	14.3
HPV16 status		
Positive	17	40.5
Negative	25	59.5

SD, standard deviation; ECOG, Eastern Cooperative Oncology Group performance status.

### Induction treatment

**Figure 1:**
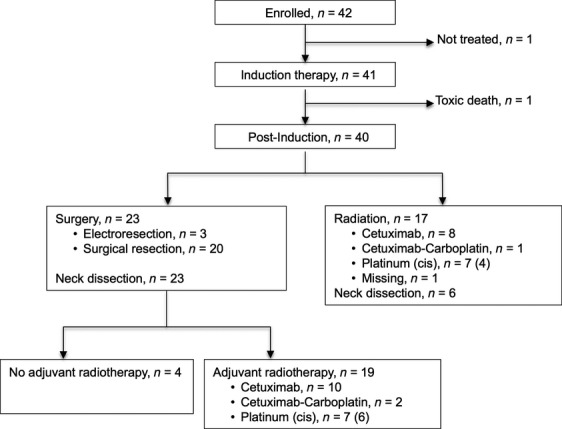
CONSORT diagram.

Forty-one patients (mITT population) started induction therapy (Fig.1). One patient did not receive an intended treatment due to investigator decision to replace cisplatin by carboplatin and not to administer cetuximab. Thirty-one (76%) patients and 36 (88%) patients received all nine planned doses of cetuximab and full three doses of planned chemotherapy, respectively. Dose reduction of cetuximab was required in two (5%) patients and in 10 (24%) patients for chemotherapy. Treatment had to be stopped early in eight (19%) patients, mainly due to limiting toxicity in five patients (diarrhea, febrile neutropenia, neutropenia without fever, diarrhea with febrile neutropenia, and skin toxicity), one toxic death (colonic perforation), one acute pancreatitis, and one consent withdrawal.

### Tumor response

After ETPF, crCR of the primary tumor at 3 months was observed in nine (22%) of 41 patients in the mITT population (Table2). Seventeen (41%) patients achieved cCR and 14 (34%) had rCR. No disease progression occurred during induction therapy. An objective response rate (ORR) of 58% was observed.

**Table 2:** Tumor response rates at 3 months in the modified intent-to-treat population (*n* = 41)

	Tumor *N* (%)	Node *N* (%)	Tumor and node *N* (%)
Clinical response (cR)			
Complete (cCR)	17 (41.5)	15 (36.6)	13 (31.7)
Incomplete	21 (51.2)	23 (56.1)	25 (61.0)
Progression	0	0	0
Not evaluable	3 (7.3)	3 (7.3)	3 (7.3)
Radiological response (rR)			
Complete (rCR)	14 (34.1)	8 (19.5)	4 (9.8)
Major partial response (≥50%)	10 (24.4)	14 (34.1)	11 (26.8)
Minor partial response (<50%)	0	3 (7.3)	3 (7.3)
Stable disease	6 (14.6)	6 (14.6)	10 (24.4)
Progression	0	0	0
Not evaluable	11 (26.8)	10 (24.4)	13 (31.7)
Clinical and radiological response (crR)			
Complete (crCR)	9 (22.0)	8 (19.5)	4 (9.8)
Incomplete	29 (70.7)	27 (65.8)	31 (75.6)
Progression	0	0	0
Not evaluable	3 (7.3)	6 (14.6)	6 (14.6)

### PFS and overall survival

**Figure 2:**
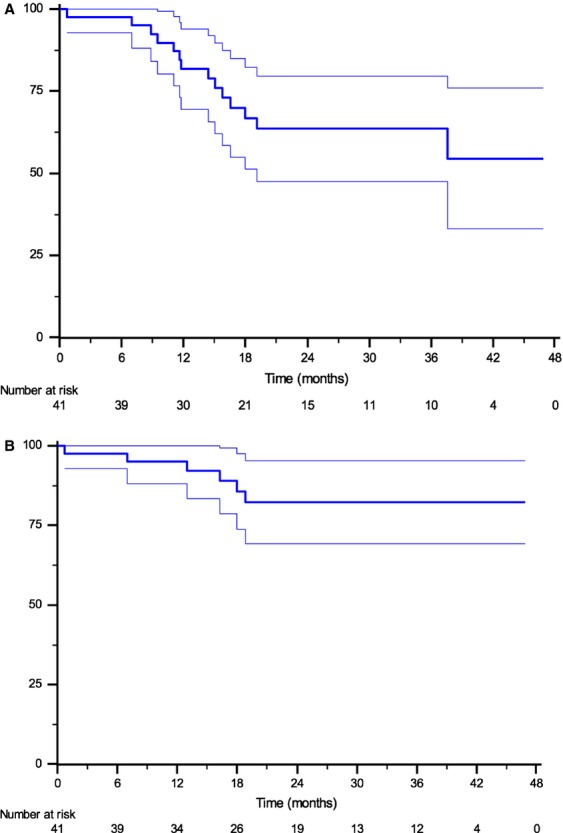
Kaplan–Meier survival curves of progression-free survival (A) and overall survival (B). Thick line defines survival curve and thin line denotes confidence boundaries placed around the true survival curve.

After a median follow-up of 23.9 months (95% CI, 15.4–28.6), median PFS was 37.6 months (95% CI, 19.1–NA), and median OS was not achieved. The 2-year estimated PFS and OS rates were 63.6% and 82.4% (standard error 8.2% and 6.6%), respectively (Fig.2). Of 11 patients with PD, three progressed locally, four progressed in nodal sites, and three had metastatic recurrence.

### Safety

The most frequent grade 3–4 toxicities in 41 treated patients were neutropenia (39%), febrile neutropenia (19%), diarrhea (10%), and stomatitis (12%) (Table3). All febrile neutropenia events occurred on days 8 or 15. Acne-like skin reactions of any grade were observed in 18 (44%) patients. One (2%) toxic death occurred from chemotherapy-induced colitis with colonic perforation during the first cycle of induction therapy. Of 18 serious AE (SAE) reported by the investigators, four were considered to be cetuximab- and 13 chemotherapy-related toxicities. None of them was unexpected.

**Table 3:** Safety evaluation carried out during induction treatment and follow-up in the modified intent-to-treat population (*n* = 41)

Adverse event	NCI-CTCAE v3.0 common toxicity criteria				
	Grade 0 *N* (%)	Grade 1 *N* (%)	Grade 2 *N* (%)	Grade 3 *N* (%)	Grade 4 *N* (%)
Hematologic toxicity					
Neutropenia	22 (53.7)	0	2 (4.9)	6 (14.6)	10 (24.4)
Anemia	7 (17.1)	26 (63.4)	7 (17.1)	0	0
Thrombocytopenia	31 (75.6)	9 (21.9)	0	0	0
Febrile neutropenia	32 (78.1)	0	0	8 (19.5)	0
Nonhematologic toxicity					
Nausea	25 (61.0)	8 (19.5)	7 (17.1)	1 (2.4)	0
Vomiting	28 (68.3)	8 (19.5)	4 (9.8)	1 (2.4)	0
Stomatitis	27 (65.8)	5 (12.2)	4 (9.8)	5 (12.2)	0
Diarrhea	14 (34.1)	12 (29.3)	11 (26.8)	3 (7.3)	1 (2.4)
Neuropathy	37 (90.2)	3 (7.3)	1 (2.4)	0	0
Acne-like skin reactions	23 (56.1)	9 (21.9)	7 (17.1)	2 (4.9)	0
Creatinine	32 (78.1)	7 (17.1)	2 (4.9)	0	0

NCI-CTC, National Cancer Institute Common Terminology Criteria for Adverse Events.

### Biomarkers analysis

Transcriptional analyses of FFPE from 38 patients were performed. Univariate analysis identified EGFRvIII mutation and EGFR amplification as predictive factors significantly correlated with rCR (Table4). Of 42 patients tested for HPV16, 17 were HPV16-positive (40%) (Table5). A crCR was observed in four (24%) and five (20%) patients with HPV-positive and HPV-negative tumor, respectively.

**Table 4:** Biomarker levels analysis according to tumor response in the patients for whom pretreatment formalin-fixed, paraffin-embedded tumor tissue block, and cryopreserved tumor blocks were available (*n* = 38)

	Response [95% CI]		All (*N *=* *38)
	Complete (*N *=* *9)	Incomplete (*N *=* *29)	
EGFR			
Median (min, max)	0.6 [0.1;2]	0.7 [0.1;89.6]	0.6 [0.1;89.6]
Mean (SD)	0.8 (0.7)	4.1 (17.1)	3.3 (14.8)
*n*	9	27	36
EGF			
Median (min, max)	0.8 [0.1;3.3]	0.7 [0.1;76]	0.7 [0.1;76]
Mean (SD)	1.1 (1.1)	3.9 (14.5)	3.2 (12.5)
*n*	9	27	36
TGF *α*			
Median (min, max)	4.2 [1.6;15.4]	4.6 [0.6;33]	4.4 [0.6;33]
Mean (SD)	5.3 (4.1)	8.2 (8.9)	7.5 (8)
*n*	9	27	36
HB-EGF			
Median (min, max)	8.8 [0.6;28.9]	7.1 [0.7;66.9]	7.5 [0.6;66.9]
Mean (SD)	9.9 (9)	15.8 (17.7)	14.3 (16)
*n*	9	27	36
BTC			
Median (min, max)	3.6 [0.9;8.6]	3.1 [0;68.3]	3.2 [0;68.3]
Mean (SD)	3.9 (2.6)	6.6 (12.9)	5.9 (11.2)
*n*	9	27	36
AREG			
Median (min, max)	0.2 [0;3]	0.2 [0;92.9]	0.2 [0;92.9]
Mean (SD)	0.7 (1.1)	9.8 (24.8)	7.5 (21.8)
*n*	9	27	36
EREG			
Median (min, max)	0 [0;0.9]	0 [0;9.5]	0 [0;9.5]
Mean (SD)	0.2 (0.3)	0.7 (1.9)	0.6 (1.7)
*n*	9	27	36
Allele 1 intron 1 CA repeats			
Median (min, max)	16 [16;20]	16 [14;20]	16 [14;20]
Mean (SD)	16.7 (1.4)	16.4 (1.4)	16.5 (1.4)
*n*	9	27	36
Allele 2 intron 1 CA repeats			
Median (min, max)	17 [16;20]	19 [15;22]	18 [15;22]
Mean (SD)	17.2 (1.4)	18.5 (2.2)	18.2 (2.1)
*n*	9	27	36
EGFRvIII mutation			
No	5 (62.5%)	23 (88.5%)	28 (82.4%)
Yes	3 (37.5%)	3 (11.5%)	6 (17.7%)
EGFR amplification			
No	3 (50%)	19 (82.6%)	22 (75.9%)
Yes	3 (50%)	4 (17.4%)	7 (24.1%)

EGF, epidermal growth factor; EGFR, epidermal growth factor receptor; TGF*α*, transforming growth factor *α*; HB-EGF, heparin-binding EGF-like growth factor; BTC, betacellulin; AREG, amphiregulin; EREG, epiregulin; AREG, amphiregulin; EREG, epiregulin; SD, standard deviation.

**Table 5:** Distribution of alcohol and tobacco use according to HPV16 status in the total study population (*n* = 42)

Use	HPV16-positive (*N*)	HPV16-negative (*N*)	All (*N*)
Alcohol only	2	1	3
Tobacco only	5	3	8
Both alcohol and tobacco	8	17	25
None	2	4	6
Total	17	25	42

HPV16, human papillomavirus type-16.

### Surgery

Neck dissection before postinduction therapy was performed in seven patients who went onto chemoradiation and in 22 patients who underwent surgery. Treatment after primary tumor resection was performed in 22 patients (Fig.1). Complete (R0) tumor resection was achieved in 17 patients.

### Chemoradiotherapy

After induction therapy, 36 patients received chemoradiotherapy, either after surgical intervention (19 patients) or without primary tumor resection (17 patients). A concomitant systemic therapy was carried out with cetuximab (18 patients), platinum salt (14 patients), or both (three patients). The median chemoradiation duration was 9.1 weeks (range, 1.0–13.1).

### Pathological response

Of 22 patients with both primary tumor resection and neck dissection, nine had a pCR of the primary tumor, and six had a pCR of both the primary and node tumors. Of the seven patients who underwent neck dissection, two had a pCR.

## Discussion

The ECHO-07 phase II study shows that addition of cetuximab to the standard TPF induction regimen in patients with locally advanced resectable stage III-IV SCC of the oropharynx produces a crCR of 22%.

The major goals of induction chemotherapy are to downsize the tumor, improve locoregional control, and target distant metastases prior to definitive treatment. TPF-based induction chemotherapy followed by chemoradiotherapy has been shown to improve outcomes (time-to-treatment failure, locoregional control) in patients with advanced SCC of the oropharynx 6,7,27. Despite the potential benefits seen in these initial studies, recent trials, DeCIDE and PARADIGM 4,5, failed to show a survival advantage with this treatment thus questioning the role of induction chemotherapy.

The ETPF regimen was previously evaluated in 50 patients with unresectable SCC of the oropharynx 28. In this phase II trial the ORR after four cycles of induction was 78%. In our study, induction ETPF was associated with a radiological tumor response (complete and partial) of 58%, a low incidence of distant metastasis (17%), and locoregional recurrence (7%). Given that the tumor response definition differs across studies of patients with SCC of the oropharynx, a comparison of response rates with those given by others 4,28,29 would be biased and misleading, therefore not acceptable. The cCR in our study is about twofold higher than crCR (41% vs. 22%). Such situation generates an urgent need to standardize the current clinical endpoints definitions and to evaluate more clinically relevant endpoints (e.g., Health related quality of life measures). Definition for the Assessment of Time-to-event Endpoints in CANcer trials (DATECAN) program to develop standardized definitions of commonly used endpoints, enabling appropriate comparisons of future trials is currently ongoing 30.

A major concern of TPF induction treatment is a high incidence of treatment-induced toxicity. In this study, febrile neutropenia during ETPF induction was reported in 19% of patients despite a systematic G-CSF support required in the protocol. This rate is higher than that reported by previous studies using TPF induction therapy (5–12%) 5–7,31, but similar to prior safety profiles when adding cetuximab to TPF 28. This may be explained by a weekly assessment of hematological toxicities, rather than the addition of cetuximab to TPF regimen. Moreover, removing 5-FU from ETPF leads to a 10% rate of febrile neutropenia 32. One (2%) treatment-related death occurred during induction therapy secondary to chemotherapy-induced colitis with colonic perforation. Of note, only 22% of SAE were considered to be cetuximab-related. 5-FU is currently a substantial part of this three-drug induction regimen, but its input remains debatable. A chemotherapy doublet induction therapy with taxanes and platinum-salt with cetuximab could be an appropriate approach to improve therapeutic index while decreasing toxicity in patients with SCC of the oropharynx 32–36. Another approach would be to use 5-FU with a shorter duration of continuous infusion as performed in other cancers 37. To reduce associated toxicity during ETPF administration, dose modifications of induction regimen might be also considered. The modified TPF regimen was shown to have similar efficacy with standard dose TPF with an acceptable toxicity profile in gastric cancer studies 38,39.

We explored the potential value of the EGFR and its ligands in predicting clinical response to ETPF treatment. Although most markers correlated positively, only EGFR amplification and EGFRvIII mutation were strongly associated with CR by univariate analysis. Recent data suggested that persistent signaling through c-MET activation in the setting of EGFR inhibition contributes to the limited clinical responses to EGFR targeting in patients with SCC of the oropharynx 40,41. Hence, future studies will need to investigate the relevance of cross talk between EGFR and c-MET signaling and define whether cosequential/sequential targeting of these oncogenic pathways may represent more effective therapy in this patient population.

HPV-positive patients with SCC of the oropharynx have a more favorable outcome compared with HPV-negative patients, however, this advantage can be obscured in heavy smokers 22. An increase in distant metastases and tumor recurrence in patients with advanced HPV-positive oropharyngeal cancer who smoked tobacco was observed 42. Moreover the risk of cancer progression/death was shown to increase directly as a function of pack-years/total number of years of smoking, regardless of HPV status 18. These findings suggest that tobacco smoking may worsen treatment response, disease control, and increase risk of developing a second primary cancer. In our study, crCR rate was comparable between HPV-positive patients (24%) and patients with HPV-negative tumors (20%). Only nine (21%) patients were nonsmokers, which indicate that the majority of treated patients were at increased risk for recurrence/death from disease. It will therefore be of great importance to stratify patients for HPV status and tobacco use in future trials to discriminate those who are at high risk for treatment failure.

In conclusion, ECHO-07 study reports an interesting response rate for ETPF in patients with moderately advanced SCC of the oropharynx. The dose levels of the ETPF combination evaluated in this study cannot be recommended. However, signs of clinical activity seen in these patients suggest that its further evaluation as induction therapy with optimal safety profile management is warranted.

## References

[b1] Ferlay J, Shin HR, Bray F, Forman D, Mathers C, Parkin DM (2010). http://globocan.iarc.fr.

[b2] Edge S, Byrd D, Compton C, Fritz AG, Greene FL, Trotti A (2010). AJCC cancer staging manual. Stomach.

[b3] Pfister DG, Ang KK, Briyzel DM, Burtness BA, Busse PM, Caudel JJ (2013). Head and neck cancers, version 2.2013. Featured updates to the NCCN guidelines. J. Natl. Compr. Canc. Netw.

[b4] Haddad R, O'Neill A, Rabinowits G, Tishler R, Khuri F, Adkins D (2013). Induction chemotherapy followed by concurrent chemoradiotherapy (sequential chemoradiotherapy) versus concurrent chemoradiotherapy alone in locally advanced head and neck cancer (PARADIGM): a randomised phase 3 trial. Lancet Oncol.

[b5] Cohen E, Karrison T, Kocherginsky M, Huang CH, Agulnik M, Mittal BB (2012). DeCIDE: a phase III randomized trial of docetaxel (D), cisplatin, 5-fluorouracil (F) (TPF) induction chemotherapy (IC) in patients with N2/N3 locally advanced squamous cell carcinoma of the head and neck (SCCHN). J. Clin. Oncol.

[b6] Vermorken JB, Remenar E, van Herpen C, Gorlia T, Mesia R, Degardin M (2007). Cisplatin, fluorouracil, and docetaxel in unresectable head and neck cancer. N. Engl. J. Med.

[b7] Posner MR, Hershock DM, Blajman CR, Mickiewicz E, Winquist E, Gorbounova V (2007). Cisplatin and fluorouracil alone or with docetaxel in head and neck cancer. N. Engl. J. Med.

[b8] Grandis JR, Tweardy DJ (1993). Elevated levels of transforming growth factor alpha and epidermal growth factor receptor messenger RNA are early markers of carcinogenesis in head and neck cancer. Cancer Res.

[b9] Chung CH, Ely K, McGavran L, Varella-Garcia M, PArker J, Parker N (2006). Increased epidermal growth factor receptor gene copy number is associated with poor prognosis in head and neck squamous cell carcinomas. J. Clin. Oncol.

[b10] Ang KK, Berkey BA, Tu X, Zhang HY, Katz R, Hammond EH (2002). Impact of epidermal growth factor receptor expression on survival and pattern of relapse in patients with advanced head and neck carcinoma. Cancer Res.

[b11] Sheridan MT, O'Dwyer T, Seymour CB, Mothersill CE (1997). Potential indicators of radiosensitivity in squamous cell carcinoma of the head and neck. Radiat. Oncol. Investig.

[b12] Chiang WF, Liu SY, Yen CY, Lin CN, Chen YC, Lin SC, Chang KW (2008). Association of epidermal growth factor receptor (EGFR) gene copy number amplification with neck lymph node metastasis in areca-associated oral carcinomas. Oral Oncol.

[b13] Karamouzis MV, Grandis JR, Argiris A (2007). Therapies directed against epidermal growth factor receptor in aerodigestive carcinomas. JAMA.

[b14] Bonner JA, Harari PM, Giralt J, Cohen RB, Jones CU, Sur RK (2006). Radiotherapy plus cetuximab for squamous-cell carcinoma of the head and neck. N. Engl. J. Med.

[b15] Vermorken JB, Mesia R, Rivera F, Remenar E, Kawecki A, Rottey S, Tam C, Perier S, Soussan P, St Guilz JL (2008). Platinum-based chemotherapy plus cetuximab in head and neck cancer. N. Engl. J. Med.

[b16] Mirghani H, Moreau F, Lefevre M, Tam C, Perier S, Soussan P, St Guilz JL (2011). Human papillomavirus type 16 oropharyngeal cancers in lymph nodes as a marker of metastases. Arch. Otolaryngol. Head Neck Surg.

[b17] Li W, Thompson CH, O'Brien CJ, McNeil EB, Scolyer RA, Cossart YE (2003). Human papillomavirus positivity predicts favourable outcome for squamous carcinoma of the tonsil. Int. J. Cancer.

[b18] Gillison ML, Zhang Q, Jordan R, Xiao W, Westra WH, Trotti A (2012). Tobacco smoking and increased risk of death and progression for patients with p16-positive and p16-negative oropharyngeal cancer. J. Clin. Oncol.

[b19] Chaturvedi AK, Engels EA, Anderson WF, Gillison ML (2008). Incidence trends for human papillomavirus-related and -unrelated oral squamous cell carcinomas in the United States. J. Clin. Oncol.

[b20] Marur S, D'Souza G, Westra WH, Forastiere AA (2010). HPV-associated head and neck cancer: a virus-related cancer epidemic. Lancet Oncol.

[b21] St Guily JL, Jacquard AC, Pretet JL, Haesebaert J, Beby-Defaux A, Clavel C (2011). Human papillomavirus genotype distribution in oropharynx and oral cavity cancer in France—The EDiTH VI study. J. Clin. Virol.

[b22] Ang KK, Harris J, Wheeler R, Weber R, Rosenthal DL, Nquyen-Tan PF (2010). Human papillomavirus and survival of patients with oropharyngeal cancer. N. Engl. J. Med.

[b23] Greene FL, Page DL, Fleming ID, Fritz A, Balch CM (2002). AJCC cancer staging manual. Exocrine pancreas.

[b24] Tanaka Y, Miyamoto S, Suzuki SO, Oki E, Yaki H, Sonoda K (2005). Clinical significance of heparin-binding epidermal growth factor-like growth factor and a disintegrin and metalloprotease 17 expression in human ovarian cancer. Clin. Cancer Res.

[b25] Sok JC, Coppelli FM, Thomas SM, Lango MN, Xi S, Hunt JL (2006). Mutant epidermal growth factor receptor (EGFRvIII) contributes to head and neck cancer growth and resistance to EGFR targeting. Clin. Cancer Res.

[b26] Etienne-Grimaldi MC, Pereira S, Magne N, Formento JL, Francoual M, Fontana X (2005). Analysis of the dinucleotide repeat polymorphism in the epidermal growth factor receptor (EGFR) gene in head and neck cancer patients. Ann. Oncol.

[b27] Hitt R, Grau JJ, Lopez-Pousa A, Berrocal A, Garcia-Giron C, Irigoyen A (2014). A randomized phase III trial comparing induction chemotherapy followed by chemoradiotherapy versus chemoradiotherapy alone as treatment of unresectable head and neck cancer. Ann. Oncol.

[b28] Mesia R, Vázquez S, Grau JJ, Garcia-Saenz JA, Bayona C, Galceran JC (2009). A single-arm phase II trial to evaluate the combination of cetuximab plus docetaxel, cisplatin, and 5-fluorouracil (TPF) as induction chemotherapy (IC) in patients (pts) with unresectable SCCHN. Int. J. Clin. Oncol.

[b29] Argiris A, Buchanan A, Brockstein B, Kolesar J, Ghebremichael M, Pins M (2009). Docetaxel and irinotecan in recurrent or metastatic head and neck cancer: a phase 2 trial of the Eastern Cooperative Oncology Group. Cancer.

[b30] Bellera CA, Pulido M, Gourgou S, Collette L, Doussau A, Kramar A (2013). Protocol of the Definition for the Assessment of Time-to-event Endpoints in CANcer trials (DATECAN) project: formal consensus method for the development of guidelines for standardised time-to-event endpoints' definitions in cancer clinical trials. Eur. J. Cancer.

[b31] Pointreau Y, Garaud P, Chapet S, Sire C, Tuchais C, Tortochaux J (2009). Randomized trial of induction chemotherapy with cisplatin and 5-fluorouracil with or without docetaxel for larynx preservation. J. Natl. Cancer Inst.

[b32] Argiris A, Heron DE, Smith RP, Kim S, Gibson MK, Lai SY (2010). Induction docetaxel, cisplatin, and cetuximab followed by concurrent radiotherapy, cisplatin, and cetuximab and maintenance cetuximab in patients with locally advanced head and neck cancer. J. Clin. Oncol.

[b33] Kies MS, Holsinger FC, Lee JJ, Jr William WN, Glisson BS, Lin HY (2010). Induction chemotherapy and cetuximab for locally advanced squamous cell carcinoma of the head and neck: results from a phase II prospective trial. J. Clin. Oncol.

[b34] Dietz A, Rudat V, Dreyhaupt J, Pritsch M, Hoppe F, Hagen R (2009). Induction chemotherapy with paclitaxel and cisplatin followed by radiotherapy for larynx organ preservation in advanced laryngeal and hypopharyngeal cancer offers moderate late toxicity outcome (DeLOS-I-trial). Eur. Arch. Otorhinolaryngol.

[b35] Guigay J, Fayette J, Dillies A, Sire J, Kerger JN, Tennevet I (2011). Cetuximab, docetaxel, and cisplatin (TPEx) as first-line treatment in patients with recurrent or metastatic (R/M) squamous cell carcinoma of the head and neck (SCCHN): first results of phase II trial GORTEC 2008-03. J. Clin. Oncol.

[b36] Wanebo HJ, Lee J, Burtness BA, Ridge JA, Ghebremichael M, Spencer SA (2014). Induction cetuximab, paclitaxel and carboplatin followed by chemoradiation with cetuximab, paclitaxel and carboplatin for stage III/IV head and neck squamous cancer: a phase II ECOG-ACRIN trial (E2303). Ann. Oncol.

[b37] de Gramont A, Bosset JF, Milan C, Rougier P, Bouche O, Etienne PL (1997). Randomized trial comparing monthly low-dose leucovorin and fluorourcail bolus with bimonthly hig-dose leucovorin and fluorourcail bolus plus continous infusion for advanced colorectal cancer: a French intergroup study. J. Clin. Oncol.

[b38] Arslan C, Koseoglu FD (2014). Modified docetaxel, cisplatin, and 5-fluorouracil combination regimen in advanced gastric cancer: Toxicity and efficacy results. J. Clin. Oncol.

[b39] Ozal G, Dogan M, Akbulut H, Yalcin B, Utkan G, Urun Y, Icli F The safety and efficacy of modified-dose docetaxel, cisplatin, and 5-fluorouracil (mDCF) combination in the front-line treatment of advanced gastric cancer.

[b40] Velpula KK, Dasari VR, Asuthkar S, Gorantla B, Tsung AJ (2012). EGFR and c-Met cross talk in glioblastoma and its regulation by human cord blood stem cells. Transl. Oncol.

[b41] Wheeler DL, Huang S, Kruser TJ, M Nechrebecki M, Armstrong EA, Benavente S (2008). Mechanisms of acquired resistance to cetuximab: role of HER (ErbB) family members. Oncogene.

[b42] Maxwell JH, Kumar B, Feng FY, Worden FP, Lee JS, Eisbruch A (2010). Tobacco use in human papillomavirus-positive advanced oropharynx cancer patients related to increased risk of distant metastases and tumor recurrence. Clin. Cancer Res.

